# Chronic traumatic ankle and foot osteomyelitis: a nationwide case-control study

**DOI:** 10.1186/s40779-018-0163-8

**Published:** 2018-05-15

**Authors:** Maryam Hosseini, Mostafa Allami, Mohammadreza Soroush, Fateme Babaha, Javad Minooeefar, Davood Rahimpoor

**Affiliations:** 1 Janbazan Medical and Engineering Research Center (JMERC), Tehran, Iran; 2 Department of Immunology, Faculty of Medical Sciences, Tarbiat Modares University, Tehran, Iran

**Keywords:** Ankle, Foot, Iran, Osteomyelitis, Quality of life, Veterans

## Abstract

**Background:**

Osteomyelitis (OM) is an atypical consequence of ankle-foot trauma which is associated with long-term mental and physical morbidity and persistent pain. This study aimed to assess the health status of OM patients with war-related ankle-foot injuries.

**Methods:**

A total of 1129 veterans with ankle-foot injuries participated in a case-control study (2014–2016). Thirty patients with chronic OM of the ankle-foot were compared with 90 non-OM participants as the control group. Quality of life (QOL), life satisfaction and the ability to perform basic and instrumental activities of daily living were measured using the following questionnaires: short-form health survey (SF-36), satisfaction with life scale (SWLS), activity of daily living (ADL) and instrumental activity of daily living (IADL), respectively. OM patients were categorized according to their risk factors as A, B and C hosts using a modified version of the Cierny and Mader classification system. The one sample *t*-test, 2-independent sample *t*-test, ANOVA, Pearson correlation coefficient and multiple linear regression analyses were applied to analyze the data.

**Results:**

Ankle-foot pain leading to surgery (*P* < 0.001) and orthosis usage (*P* = 0.039) were more common in OM patients. There was no significant difference between the two groups in the prevalence of pulmonary and cardiovascular diseases or kidney failure and other related diseases. OM patients showed a significantly lower level of mental health compared to non-OM respondents (*P* = 0.025). Approximately, 70.0% of ankle-foot injured veterans were dissatisfied with their life, and there was no difference between the two groups (*P* > 0.05). Mobility was significantly lower in the OM patients than in the control group (*P* = 0.023). Life satisfaction (*P* = 0.001) and the ability to perform daily activities were the determinants for poor physical (*P* = 0.018) and mental (*P* = 0.012) health-related quality of life. According to the Cierny and Mader classification system, they were all included in the type C host classification, with one major and/or three or more minor risk factors.

**Conclusions:**

A low level of quality and satisfaction of life and ability to perform activities of daily living were observed in OM patients with war-related ankle-foot injuries. Surgeries of the ankle and foot due to pain were much more common in OM patients than in non-OM participants. Since all the participants were classified as the C-host, health policy planning seems to be necessary.

## Background

Osteomyelitis (OM) is a bone infection that originates from different sources: hematogenous spread, direct inoculation (due to an open fracture or surgery) or contiguous spread (due to an adjacent soft tissue infection) [1]. Bone infection manifests as several clinical signs, such as redness, heat, swelling and ulcer discharge [2]. Antibiotic administration and surgery are the leading treatment approaches for OM [3]. However, many studies have described that infections resistant to antibiotics blocks full remission of the disease [2, 4, 5]. Therefore, recurrence seems to be an ongoing obstacle that results in a 30% therapeutic failure rate [4], losing the hope for successful treatment. Many studies have demonstrated the association between chronic illness and increased mental and psychological problems [5–7]. Psychological adjustment to a disease is as important as the physical treatment of the condition [5].

Given that OM imposes heavy physical difficulties and permanent periodic medical and surgical treatment, patients face subsequent mental health issues that directly affect their social and personal lives [8]. Persistent pain is considered to be one of the main factors to poor quality of life [6]. Physical limitations imposed by pain influence the performance of daily tasks for these patients and, in some cases, can result in semi- or full dependency. Furthermore, pain generates resistance to psychological adjustment, and it is considered to be the major reason for complaints among chronic OM patients [9]. Physical burden derived from OM and ongoing reliance on medical therapies require social support, strong mental integrity and the ability to cope with a variety of complications that caused by the disease, such as altered sexuality, mobility limitations and other orthopedic problems. The absence of effective coping skills in OM patients may lead to depression, overall dissatisfaction with life, drug addiction and maladaptive behaviors [8].

Unlike diabetic and postoperative patients, there is insufficient literature evaluating veterans with post-traumatic OM due to battlefield injuries. Specific research on Iraq war victims from American military operations demonstrated that OM was a common complication [10]. Musculoskeletal injuries comprised more than 70% of all war-related wounds [11]. Detonation and blasts from landmines and other explosive weapons were the main underlying reasons for orthopedic injuries and bone fractures [12]. Injuries to the upper and lower extremities comprised two-thirds of all war-related wounds, which were often characterized by open fractures [13]. Wound contamination at the time of injury, early aggressive treatment in the battlefield and insufficient facilities to treat injured soldiers lead to further complications [10]; therefore, individuals with war-related injuries encounter various secondary complications [14]. In the three decades since the Iran-Iraq war, more than 500 thousand veterans with different types of injuries were registered in the Veterans and Martyrs Affair Foundation (VMAF), 55% of whom suffered from musculoskeletal disorders of the lower extremities [13]. Among veterans with lower extremity injuries, OM cases comprise a small proportion of all of these injuries but tend to have more complications due to severe conditions [15]. In musculoskeletal injuries, limb salvage is the primary treatment approach for orthopedic surgeons who specialize in war injuries. This amputation-sparing treatment left patients with serious limb deformities for years afterwards and required the ongoing need for medical and surgical treatment. OM is considered an uncommon secondary disorder in veterans with musculoskeletal injuries. Three major complications these patients experience represent their main complaints: 1) continuous malodorous discharge from the infected limb that makes it difficult for patients to remove their shoes in public places and forces them to intermittently change orthoses or medical shoes; 2) deformities of the lower extremity that cause mobility complications; and 3) persistent pain that leads to negative emotions [8].

As a result of the Iran-Iraq war, many civilians and military personnel sustained severe injuries. In the long term, these survivors suffer from various war-related disorders as a consequence of their former injuries. Over the last thirty years, this population has aged or are entering into the later stages of life. Even normal individuals are susceptible to chronic illnesses and related psychological distress; thus, aging patients with active OM and coexisting physical disorders face worse psychological and physical conditions [6].

To study the health status of chronic OM patients, we enrolled a group of patients with war-related ankle-foot injuries who developed OM as case studies and compared them with other patients with ankle-foot injuries who did not have OM. To our knowledge, this is the first study to evaluate the general health and Quality of life (QOL) associated factors in this cohort. In the present study, we discussed the dependency, life satisfaction, overall mental and physical status of these individuals and the major risk factors for worsening OM. This study aimed to provide insight into better management of this illness and suggest adequate measures for improving the QOL of OM patients.

## Methods

### Study design

This case-control study was performed during 2014–2016. The lists and information of participants were obtained from the VMAF databank. According to the data, a total of 10,227 veterans with war-related ankle and foot injuries were found across the country. Using random sampling, veterans from 11 provinces were invited to participate in the study. After three years, 1129 veterans with ankle-foot disorders were visited by scientific teams, including orthopedists, internists, prosthetists and orthotists. Orthotic/prosthetic rehabilitation plays a key role in physical activity improvement, particularly in walking, for people who suffer from lower limb impairments. Disruption of the peripheral nerves of the lower extremities leads to impaired joint function. Loss of ankle joint control generates various difficulties during walking, such as drop foot. In this cohort, the increased risk of falling while climbing or descending on uneven terrain was confirmed in previous studies [16, 17]. The use of ankle-foot orthoses (AFO) significantly decreases the frequency of falls [18]. In many other cases, the small foot joints are fused due to fragment wounds from explosions. Soft, custom-made insoles alleviate pain by reducing the sole parts that are under pressure. In addition, limping while walking results from leg length discrepancy (LLD) in more than half of the study group, which causes pain and damage to the spine [19]. Insoles that provide additional height can compensate for LLD, and the secondary effects can mostly be eliminated [20]. The most common prescribed orthoses for both OM and non-OM participants consist of medical shoes, insoles with LLD compensation, soft knee supports, soft insoles, AFOs, and shoe modifications, such as lateral and medial wedges, flares, bars and pads. There are a variety of materials used to construct insoles, including foam, leather, metal, and plastic.

According to the inclusion criteria, veterans who suffered from neuromusculoskeletal problems in the ankle and/or foot due to following reasons were enrolled: 1) ankle and/or foot trauma and partial amputation due to a hit from direct bullet or fragments; 2) leg or thigh trauma that led to neuromusculoskeletal disorders of the ankle and foot; and 3) neuromusculoskeletal damage of the lower extremity leading to dysfunction of the ankle and/or foot that was managed with ankle-foot orthotics. Patients with amputations at the level of the ankle or higher or patients who had central nervous system damage were excluded. The exclusion criteria also include patients who were suffering from contralateral lower limb injuries or amputations. Of 1129 participants, thirty who suffered from chronic OM of the ankle and/or foot were enrolled as the case group. A group of 90 non-OM participants was selected for the control group using random sampling from the group of 1099 patients who remained after the OM patients were selected. The case and control groups were matched based on age, gender, disability rate and province. Informed consent was obtained from all the veterans. The study was supported by VMAF and Janbazan Medical and Engineering Research Center (JMERC). Trained experts interviewed the veterans and completed the forms. Demographic data included age, gender, employment, disability rate, educational and marital status and other injuries. Data including hospitalization records, orthosis usage and pain that led to surgery were also collected. Body mass index (BMI) was measured using height and weight observations and calculated by taking the weight in kilograms divided by the square of the height in meters.

### Study tools

To evaluate the participants’ quality of life, a short-form health survey (SF-36) questionnaire was utilized [21]. SF-36 consists of 36 questions assessing eight health-related concepts in a multi-item scale format including the following: 1) physical functioning (PF); 2) social functionality (SF); 3) limitations of daily activities as a result of a physical problem (RP); 4) bodily pain (BP); 5) overall mental health (MH); 6) limitations of activities following emotional problems (RE); 7) vitality (VI); and 8) general health perception (GH). SF-36 is a generic tool constructed to evaluate the physical and mental health of the person (Physical Component Scale, PCS and Mental Component Scale, MCS). Montazeri et al. [22] validated the Persian version of this questionnaire in 2005 in a normal Iranian population. The internal consistency showed the reliability standard, and the Cronbach’s alpha coefficients ranged from 0.77 to 0.90.

Life satisfaction of the veterans was measured by the satisfaction with life scale (SWLS) questionnaire. SWLS includes five general questions that evaluate an individual’s subjective well-being in reference to their own criteria and perceptions. In this self-judgmental process, the person assesses his/her own life on the basis of their own self-imposed standards. The questions are rated on a 7-point Likert scale ranging from “totally disagree” to “totally agree” (1 to 7). The overall score ranges from 5 to 35. SWLS was validated (Cronbach’s alpha = 0.83) by Bayani et al. [23].

To assess the functional status of the participants, the activity of daily living (ADL) instrument known as Barthel’s index was used [24]. This questionnaire measures personal performance on 10 grounds: bathing, grooming, dressing, feeding, toileting, transfer, walking, bowels and bladder continence and using stairs. The test evaluates an individual’s ability in performing the abovementioned tasks. Regarding the participant’s dependencies, the answers are scored differently for each item and range from zero to 15 to address whether the person can do the activity on their own (highest score) or whether they need assistance from another person or the use of special instruments or mechanical aids. The overall score can reach a maximum of 100 or a minimum of 0 to indicate fully independent and dependent, respectively. Cronbach’s alpha for the ADL instrument was reported to be as high as 0.96.

More complex sets of activity used in daily living are described by the instrumental activity of daily living (IADL) measure, which had a Cronbach’s alpha coefficient of 0.81. Compared to the activities described by ADL, these activities require more complex human behavior to cope with the environment. They include adaptive daily tasks, such as the use of the telephone, shopping, cooking, housekeeping, laundry, the use of transportation, managing money, and managing medications [25]. IADL ability is scored on a 3-point scale (dependent: 0, need help:1 and independent: 2). Self-maintenance or dependents of individuals rated the answers ranging from “very dependent” to “independent” (0 to 16).

To classify the patients according to their risk factors, a modified version of the Cierny and Mader classification system was used [26]. The original Cierny and Mader classification system is a clinical staging system for OM in adults that categorizes patients into four anatomic types (disease) and three physiologic classes (hosts) to describe 12 clinical stages. This categorization provides a guideline for treatment approaches and is effective for comparing the results from different treatment protocols [27]. According to this classification, the A and B hosts are designated for curative treatment protocols, whereas the C hosts are those who are not treatment candidates because the treatment or its results compromise the host more than the disease itself; therefore, these hosts receive palliation. In this stratification, the physiological status of the hosts, particularly the C host, seems subjective and problematic because it is highly dependent on the treating surgeon’s experience. The differentiation between B and C hosts is followed by a decision regarding a choice between a palliative or curative approach that requires more precise and objective criteria [26]. For the physiological status, a modified version of the Cierny and Mader classification system provides a more pragmatic definition of hosts. Hosts are categorized according to associated risk factors; type A hosts have no risk factors; type B hosts have less than three minor risk factors; and type C hosts have one major and/or three or more minor risk factors [28]. Additionally, the internists (one in each province) evaluated all the participants for the presence of pulmonary and cardiovascular diseases and kidney failure and other related diseases.

### Statistical analysis

Statistical analysis was performed using SPSS 16.0 (The Statistical Package for the Social Sciences, version 16.0, SPSS Inc., Chicago, IL, USA). Quantitative variables were reported as the mean ± standard deviation, and qualitative variables were presented as frequency and percent. The patients’ scores on the SF-36 were compared with those of the general Iranian population using a one sample *t*-test [22]. The relationships between the quantitative variables, such as ADL/IADL and life satisfaction, with the MCS and PCS scores were examined by Pearson’s correlation coefficient. To compare the variables between the two case and control groups, the 2-independent sample *t*-test and ANOVA were applied. We performed multiple linear regression analyses to determine the variables that contributed most to health-related quality of life in veterans with ankle-foot trauma. The PCS and MCS were used as dependent variables. The variables that showed a significant *P* value were entered in the regression model. *P* values < 0.05 were considered significant.

## Results

The mean age of the participants was 51.73 ± 7.66 years, and the average disability rate was 35.88 ± 12.83. All the participants were married (100.0%). Approximately half of the patients in each group had an education level of “less than diploma”. They were mostly unemployed or retired (Table 1). War-related injuries other than ankle-foot injuries were observed in approximately 50% of patients.

**Table 1:** Sociodemographic characteristics of veterans with ankle-foot disorders

Characteristics	Case group (*n* = 30)		Control group (*n* = 90)		*P* value
	Frequency	Percent (%)	Frequency	Percent (%)	
Age groups					
Less than 34 years	1	3.3	1	1.1	0.668
35 to 44 years	5	16.7	8	8.9	
45 to 54 years	15	50.0	57	63.3	
55 to 64 years	7	23.3	18	20.0	
65 years and older	2	6.7	6	6.7	
Mean ± SD	51.20 ± 8.91		51.90 ± 7.24		
Disability rates					
25 to 49%	24	80.0	72	80.0	0.913
50 to 69%	5	16.7	15	16.7	
70%	1	3.3	3	3.3	
Mean ± SD	36.17 ± 13.24		35.78 ± 12.76		
Education					
Illiterate	2	6.7	6	6.7	0.923
Less than diploma	14	46.7	33	36.7	
Diploma	7	23.3	33	36.7	
University studies	8	26.7	18	20.0	
Gender					
Male	29	96.7	87	96.7	1.000
Female	1	3.3	3	3.3	
Employment					
Employed	8	26.7	17	17.9	0.159
Unemployed	4	13.3	3	3.3	
Employment status	11	36.7	34	37.8	
Retired	7	23.3	36	40.0	
Other injuries					
Yes	13	46.7	41	45.6	0.668
No	17	53.3	55	54.4	

Participants who suffered from OM received significantly more orthoses after they were injured (*P* = 0.039). In addition, the prevalence of ankle pain that led to surgery in these patients was doubled (*P* < 0.001). Chronic joint pain was significantly more common in the non-OM group (*P* = 0.048) (Table 2). Musculoskeletal disorders were observed in approximately three-quarters of the participants in both groups. There was no significant difference between the two groups in the prevalence of pulmonary and cardiovascular diseases and kidney failure and other related diseases (data not shown). The BMI values were significantly different between both groups (*P* = 0.012); the average BMI in the case and control groups was 25.99 ± 3.81 kg/m^2^ and 28.64 ± 5.25 kg/m^2^, respectively. Classification of the OM patients according to their risk factors showed that all except one had at least one major risk factor, including active cellulitis or abscess formation (96.7%). The minimum number of minor risk factors observed was two (26.7%), and the maximum number was estimated to be six (3.3%). As specified by the modified version of the Cierny and Mader classification system, all of the patients (100.0%) were classified as the type C-host (one major and/or three or more minor risk factors).

**Table 2:** Other demographic characteristics of veterans with ankle-foot disorders [*n*(%)]

Characteristics	Case group (*n* = 30)		Control group (*n* = 90)		*P* value
	Yes	No	Yes	No	
Hospitalization regardless of the cause of injuries	5 (16.7)	25 (83.3)	16 (17.8)	74 (82.2)	0.910
Hospitalization due to war-related injuries	9 (30.0)	21 (70.0)	17 (18.9)	73 (81.1)	0.196
Sick leave	0 (0.0)	30 (100.0)	5 (5.5)	85 (94.5)	0.083
Orthosis usage until the study	17 (56.7)	13 (43.3)	33 (36.7)	57 (63.3)	0.039
Orthosis usage at the time of the study	8 (26.7)	22 (73.3)	18 (20.0)	72 (80.0)	0.685
Lower limb pain that led to surgery					
Hip	5 (16.7)	25 (83.3)	20 (22.2)	70 (77.8)	0.227
Knee	10 (33.3)	20 (66.7)	25 (27.8)	65 (72.2)	0.534
Ankle-foot	23 (76.7)	7 (23.3)	33 (36.7)	57 (63.3)	< 0.001
Back pain that led to surgery					
Neck	2 (6.7)	28 (93.3)	1 (1.1)	89 (98.9)	0.090
Thoracic	0 (0.0)	30 (100.0)	0 (0.0)	90 (100.0)	1.000
Back	1 (3.3)	29 (96.7)	5 (5.6)	85 (94.4)	0.696
Sacral	1 (3.3)	29 (96.7)	1 (1.1)	89 (98.9)	0.435
Chronic joint pain	14 (46.7)	16 (53.3)	52 (57.8)	38 (42.2)	0.048
Musculoskeletal disorders	23 (76.7)	7 (23.3)	64 (71.1)	26 (28.9)	0.535

The significant differences between the SF-36 results of the OM patients and the normal population in all eight dimensions (*P* < 0.001) indicate a poor quality of life for people who suffer from OM of the ankle-foot. The same result was obtained in the MCS in the comparison of the two groups of participants (*P* = 0.025). However, compared to the control group, the OM patients had lower mean scores in most dimensions (Table 3).

**Table 3:** SF-36 quality of life scores in veterans with ankle-foot injuries

Item	Case group (*n* = 30)	Control group (*n* = 90)		Iranian general population (*n* = 1997)	
	Mean ± SD	Mean ± SD	*P* value*	Mean ± SD	*P* value*
Physical functioning (PF)	42.20 ± 23.11	42.83 ± 22.31	0.949	85.3 ± 20.8	< 0.001
Role physical (RP)	15.83 ± 23.20	25.00 ± 33.10	0.292	70.0 ± 38.0	< 0.001
Bodily pain (BP)	24.42 ± 23.83	24.08 ± 18.80	0.874	79.4 ± 25.1	< 0.001
General health (GH)	34.00 ± 20.53	33.50 ± 19.24	0.362	67.5 ± 20.4	< 0.001
Vitality (VT)	44.67 ± 21.99	49.83 ± 20.21	0.261	65.8 ± 17.3	< 0.001
Social functioning (SF)	41.25 ± 27.49	47.36 ± 26.20	0.244	76.0 ± 24.4	< 0.001
Role emotional (RE)	18.89 ± 32.38	36.67 ± 43.00	0.050	65.6 ± 41.4	< 0.001
Mental health (MH)	46.53 ± 25.59	50.93 ± 19.71	0.362	67.0 ± 18.0	< 0.001
PCS	29.10 ± 15.77	31.35 ± 16.00	0.503	–	–
MCS	37.83 ± 21.50	46.20 ± 20.40	0.025	–	–

- Not reported, *PCS* Physical component scale, *MCS* Mental component scale

*.Compared with case group

The mean scores for life satisfaction in both the case and control groups were assessed to be less than half of the total score. According to the results in Table 4, more than two-thirds (70.0%) of the veterans with ankle-foot injuries were dissatisfied with their lives, and the average numbers were not significantly different between the two groups.

**Table 4:** Life satisfaction in veterans with ankle-foot injuries

Life satisfaction levels	Case group (*n* = 30)		Control group (*n* = 90)	
	Frequency	Percent(%)	Frequency	Percent(%)
Very satisfied	1	3.3	6	6.7
Satisfied	2	6.7	8	8.9
Moderately satisfied	6	20.0	13	14.4
Slightly dissatisfied	9	30.0	20	22.2
Dissatisfied	7	23.3	23	25.6
Very dissatisfied	5	16.7	20	22.2
Total	30	100.0	90	100.0
Mean ± SD	16.28 ± 6.82		16.27 ± 7.49	
*P* value	0.816			

With regard to subjects’ ability to perform activities of daily living, bowels (96.7%) and feeding (93.3%) were the ones which the majority of subjects were independent in. On the other hand, using stairs was the activity that the participants were able to perform the least independently (20.0–24.4%). Mobility was the only activity in which OM patients were significantly more dependent than the control group (*P* = 0.023). The IADL results for each activity and for the total mean showed no significant differences between the two groups (Table 5).

**Table 5:** ADL and IADL abilities in veterans with ankle-foot injuries (*N* = 120)

Activities	Case group (*n* = 30)				Control group (*n* = 90)				*P* value
	Independent [n(%)]	Need help [n(%)]	Dependent [n(%)]	Mean ± SD	Independent [n(%)]	Need help [n(%)]	Dependent [n(%)]	Mean ± SD	
ADL									
Feeding	28 (93.3)	2 (6.7)	0(0.0)	9.67 ± 1.27	84 (93.3)	5 (5.6)	1 (1.1)	9.61 ± 1.55	0.977
Bathing	20 (66.7)	10 (33.3)	0(0.0)	3.33 ± 2.40	70 (77.8)	20 (22.2)	0(0.0)	3.86 ± 2.11	0.251
Grooming	26 (86.7)	4 (13.3)	0(0.0)	4.33 ± 1.73	74 (82.2)	16 (17.8)	0(0.0)	4.10 ± 1.93	0.558
Dressing	22 (73.3)	7 (23.3)	1 (3.3)	8.50 ± 2.67	67 (74.5)	21 (23.3)	2 (2.2)	8.60 ± 2.50	0.907
Toilet use	24 (80.0)	4 (13.3)	2 (6.7)	8.67 ± 2.92	74 (82.2)	14 (15.6)	2 (2.2)	8.99 ± 2.28	0.737
Bowels	29 (96.7)	0(0.0)	1 (3.3)	9.67 ± 1.83	83 (92.2)	2 (2.2)	5 (5.6)	9.33 ± 2.41	0.401
Bladders	23 (76.7)	6 (20.0)	1 (3.3)	8.67 ± 2.60	71 (78.9)	15 (16.7)	4 (4.4)	8.71 ± 2.67	0.851
Transfer	23 (76.7)	7 (23.3)	0(0.0)	12.86 ± 3.95	62 (68.9)	28 (31.1)	0(0.0)	12.95 ± 3.27	0.737
Mobility	17 (56.7)	7 (23.3)	6 (20.0)	10.67 ± 5.98	71 (78.9)	10 (11.1)	9 (10.0)	12.84 ± 4.72	0.023
Using Stairs	6 (20.0)	17 (56.7)	7 (23.3)	4.83 ± 3.34	22 (24.4)	50 (55.6)	18 (20.0)	5.22 ± 3.36	0.579
Sum				81.43 ± 19.38				84.42 ± 13.92	0.797
IADL									
Using telephone	25 (83.3)	4 (13.3)	1 (3.3)	1.80 ± 0.48	84 (93.3)	6 (6.7)	0(0.0)	1.93 ± 0.25	0.098
Shopping	12 (40.0)	8 (26.7)	10 (33.3)	1.07 ± 0.87	40 (44.4)	24 (26.7)	26 (28.9)	1.15 ± 0.85	0.661
Food preparation	3 (10.0)	10 (33.3)	17 (56.7)	0.53 ± 0.68	19 (21.1)	33 (36.7)	38 (42.2)	0.80 ± 0.77	0.102
Housekeeping	5 (16.7)	8 (26.7)	17 (56.7)	0.60 ± 0.77	15 (16.7)	32 (35.6)	43 (47.8)	0.70 ± 0.74	0.476
Laundry	6 (20.0)	13 (43.3)	11 (36.7)	0.83 ± 0.75	13 (14.4)	37 (41.1)	40 (44.4)	0.71 ± 0.71	0.417
Transportation	16 (53.3)	11 (36.7)	3 (10.0)	1.43 ± 0.68	51 (56.7)	34 (37.8)	5 (5.6)	1.51 ± 0.60	0.669
Responsibility for medications	19 (63.3)	10 (33.3)	1 (3.3)	1.60 ± 0.56	63 (70.0)	21 (23.3)	6 (6.7)	1.63 ± 0.61	0.626
Handle finances	20 (66.7)	8 (26.7)	2 (6.7)	1.60 ± 0.62	72 (80.0)	12 (13.3)	6 (6.7)	1.73 ± 0.58	0.168
Sum				9.47 ± 3.73				10.15 ± 3.01	0.317

*ADL* Activity of daily living, *IADL* Instrumental activity of daily living

To enter the variables into the regression model, the significant relationships between the quantitative variables and MCS/PCS were assessed, and the outcomes are described in Table 6. MCS and PCS did not show a significant difference in accordance with educational level (*P* = 0.707 and 0.862, respectively) and employment status (*P* = 0.062 and 0.719, respectively). The results obtained from the multiple linear regression analyses showed that life satisfaction, ADL and IADL (*P* < 0.001) were predictive factors for poor physical health-related quality of life (Table 7). The analysis also showed that life satisfaction and ADL (*P* < 0.05) were the determinants of poor mental health in veterans with ankle-foot injuries.

**Table 6:** Relationships between the variables with PCS and MCS in veterans with ankle-foot injuries

Variables	PCS		MCS	
	*P* value	*r*	*P* value	*r*
Age	0.141	−0.135	0.465	0.067
Disability rate	0.015	−0.221	0.567	−0.053
ADL	< 0.001	0.467	< 0.001	0.414
IADL	< 0.001	0.412	< 0.001	0.377
Life satisfaction	< 0.001	0.427	< 0.001	0.463
BMI	0.052	−0.183	0.807	− 0.023

*PCS* Physical component scale, *MCS* Mental component scale, *ADL* Activity of daily living, *IADL* Instrumental activity of daily living, *BMI* Body mass index, *r* Correlation coefficient

**Table 7:** Determinants of the physical and mental components of quality of life in veterans with ankle-foot injuries

Determinants	*B*	Std. Error	Beta	*t*	Sig.
PCS					
Constant	−16.737	7.115	–	−2.352	0.021
Disability rate	−0.182	0.101	−0.146	−1.798	0.075
Life satisfaction	0.615	0.181	0.280	3.404	0.001
ADL	0.267	0.111	0.256	2.407	0.018
IADL	1.114	0.543	0.215	2.053	0.043
MCS					
Constant	−11.185	9.176	–	−1.219	0.226
Life satisfaction	1.021	0.238	0.364	4.292	< 0.001
ADL	0.358	0.141	0.268	2.543	0.012
IADL	0.774	0.706	0.117	1.096	0.276

*B.* Regression coefficient, *PCS* Physical component scale, *MCS* Mental component scale, *ADL* Activity of daily living, *IADL* Instrumental activity of daily living, − No data

## Discussion

In a study of 1129 survivors with traumatic ankle-foot disorders, OM was diagnosed in less than 0.03%. The complication rates of superficial and deep wound infections of acute ankle and foot fractures have been reported to be 3 to 40% [29–31]. To our knowledge, this is the first report of long-term OM incidence in war-related injuries. According to the results, the entire population of veterans with ankle-foot injuries (OM and non-OM) had lower QOL in the eight dimensions of health compared to the normal Iranian population [22]. In the intergroup comparisons, role emotional and mental components were significantly lower in OM patients. Even without a statistical difference between the two groups in the life satisfaction results, both the OM and non-OM participants had a mean score below half of the maximum satisfaction. These results concur with previous data obtained from Iranian survivors with various kinds of injuries [32–35]. Similar to the long-term study of Australian survivors after the Korean War, life satisfaction and quality of life in those veterans was reported to be poor relative to other Australian men, and combat severity was the main factor associated with this decrease in satisfaction and quality of life [36]. Comparing QOL in patients with chronic refractory OM of the lower extremities with patients who underwent amputations, it was reported that further pain and reduced social activity, ADL and mobility were more common in OM patients, but their physical activity was lower than that of amputees [8].

In the report of the ability to perform ADL, the mean score of mobility in OM patients was significantly lower than that of the non-OM group. Although the average score of transfer was not significantly different between the two groups, OM patients showed a lower transfer ability compared with the non-OM respondents. Our field observations indicated that pain was the dominant cause of decreased mobility and transfer in the OM group. This finding was in agreement with the finding of the higher prevalence of chronic joint pain in non-OM participants. As a matter of fact, those who used their joints more manifested chronic joint pain at a higher rate. In contrast to our expectations, BMI was lower in OM patients who reported lesser mobility and transfer, and this finding was statistically significant when compared with non-OM patients. In contrast to the current results, we predicted more kidney failure, pulmonary and cardiovascular diseases in OM patients as a consequence of decreased mobility and increased antibiotic use. Further studies on the general health of this cohort would be informative to provide a clearer explanation of the present results.

Injured veterans age on average one decade younger than the normal Iranian population [37]. Most of the participants were in the sixth decade of life, and they had already begun an elderly lifestyle. Apart from their elderly age, the associated injuries that were reported in approximately half of the participants were a second cause of decreased quality of life. As specified by the OM patients’ risk factors, the majority of these patients suffered from at least one major plus two or more minor risk factors. As a result, none of the OM patients were categorized as A or B-hosts and all of them were classified as C-hosts, mostly due to other war-related injuries. The failure rate for the management of chronic non-traumatic OM was reported to be as high as 20% [38], and as time passed, infections remained difficult to cure using antibiotic therapy [39]. With one exception, all OM patients suffered from active OM, which indicates a failure of treatment throughout the three decades after the war ended. In addition, the twofold prevalence of ankle-foot surgery due to pain in OM patients compared with the non-OM participants who had similarly suffered from ankle-foot disorders for a long time can be considered a confirmation of treatment failure. Conversely, the use of orthoses among OM patients was more common than in non-OM patients. According to our orthotic experts, these patients were prescribed a variety of orthoses to decrease their pain. A less common reason was orthosis deformation as a result of discharge.

For the first time, we reported quality of life and its determinants, including life satisfaction and the ability to perform ADL/IADL, in war survivors with ankle-foot disorders that were grouped and compared based on their presentation with OM. Although the number of patients in this study was limited, which suggests that further study is needed, it was a nationwide study that drew a sample from across the country. The second limitation was the unavailability of OM patients for follow-up examinations and further assessment. Beginning with the aging period, a number of major and minor risk factors and a two-to-three times lower QOL of OM patients compared with the normal population reveal the importance of studying and making healthcare policies for this study group. In fact, nations with large veteran populations may need to consider the predictive factors of QOL that can assist in helping veterans who suffer from chronic pain and long-term health problems to cope with aging. It is worth noting that the OM patients’ condition also influenced their family members’ quality of life.

## Conclusions

These results illustrated that the QOL of veterans with ankle-foot injuries was significantly lower than the normal Iranian population, and those who also suffered from OM had a lower level of mental health than the control group. According to the major and minor risk factors, all the OM patients were classified as type C hosts, and considering their mean age, they can be considered to be a group of veterans who have a need for health policy planning. In spite of the fact that no significant difference was observed between the case and control groups in life satisfaction, the mean score of this assessment in all the participants indicated their dissatisfaction. Mobility was the only task in basic and instrumental daily activities that was significantly lower in OM patients. Additionally, the OM group underwent surgeries of the ankle and foot due to pain approximately two times more frequently than the non-OM group, indicating that pain was one of their major problems. They utilized orthoses more commonly than the control group, which was likely a result of the difficulty of finding a suitable orthotic to lessen their pain.

## Abbreviations

ADL: Activity of daily living
AFO: Ankle foot orthoses
BMI: Body mass index
BP: Bodily pain
GH: General health
IADL: Instrumental activity of daily living
JMERC: Janbazan Medical and Engineering Research Center
LLD: Leg length discrepancy
MCS: Mental component scale
MH: Mental health
OM: Osteomyelitis
PCS: Physical component scale
PF: Physical functioning
RM: Role emotional problem
RP: Role physical problem
SF: Social functionality
SF-36: Short form health survey
SWLS: Satisfaction with life scale
VI: Vitality
VMAF: Veterans and Martyrs Affair Foundation
